# Optimization of the basophil activation test in the diagnosis and qualification for treatment by means of allergen-specific immunotherapy in children with respiratory allergy to house dust mites

**DOI:** 10.1186/2045-7022-5-S1-P10

**Published:** 2015-03-11

**Authors:** Radoslaw Spiewak, Aleksandra Gregorius, Ewa Czarnobilska

**Affiliations:** 1 Jagiellonian University Medical College Faculty of Pharmacy, Dept of Experimental Dermatology and Cosmetology, Krakow; Poland; 2 Jagiellonian University Medical College Faculty of Pharmacy, Department of Toxicology and Environmental Disease, Krakow, Poland

## 

Diagnosis of diseases, especially qualification for treatment should be based on the methods properly optimized in order to minimize the risk of incorrect qualification to unnecessary treatments, or neglecting treatment wherever it is needed. Among possible alternatives for routine allergy diagnostic tests, hopeful mentions can be found of the basophil activation test (BAT), which replicates in vitro reactions that occur in the body during basophil response to allergens, thus giving a chance for forecasting the severity of allergic reaction.

## Aim

The aim of the study was to optimize the basophil activation test in the detection of house dust mite allergy in Polish children with allergic respiratory diseases.

## Patients and methods

The study involved 32 patients, with symptoms of asthma or allergic rhinitis, qualified for sIT with the *D pteronyssinus* allergen by an experienced pediatric allergist. The control group consisted of an equal number of sex- and age-matched children with the same clinical diagnoses, yet sensitized to allergens other than *D. pteronyssinus*. In all patients, the BAT test was performed with five dilutions of *D. pteronyssinus* allergen. In order to detect possible cross-reactivity, BAT was also carried out with one dilution of *D. farinae*. The results were analyzed by the means of ROC.

## Results

The highest diagnostic efficiency in the analyzed population was yielded by the cut-off of 9.76% of activated basophils after activation with a single allergen concentration of 2.25 ng/ml (sensitivity 90,63%, specificity 100%). The computed value differed significantly from the cut-off value of 15% proposed by the manufacturer of the test. Qualification of the patients with the use of the proposed protocol and cut-off value did not differ from the "gold standard", i.e. qualification by the physician, while adoption of the parameters proposed by the manufacturer would result in a significant difference in this regard.

## Conclusions

BAT is a useful method in the diagnosis and qualification of children with asthma and allergic rhinitis for the allergen-specific immunotherapy with house dust mite allergens. Optimal cut-off values for the test differ from those proposed by the manufacturer of the reagents. Facing the risk of incorrect qualification of patients for costly, long-lasting and potentially risky allergen-specific immunotherapy, incurring the cost of basophil activation test seems justified, especially in the case of diagnostic doubts.

